# Health in Elite Sports from a Salutogenetic Perspective: Athletes' Sense of Coherence

**DOI:** 10.1371/journal.pone.0102030

**Published:** 2014-07-11

**Authors:** Jochen Mayer, Ansgar Thiel

**Affiliations:** Institute of Sports Science, University of Tübingen, Tübingen, Germany; City University of Hong Kong, Hong Kong

## Abstract

**Objective:**

Considering the high number of stressors encountered in the context of elite sports, a high sense of coherence (SOC) is crucial to allow athletes to maintain their health from both short- and long-term perspectives. The aim of this cross-sectional study was to investigate SOC in a population of elite athletes, focusing on identification of subsets of athletes with particularly high and low SOC scores, and any related predictors. The elite athletes' SOC scores were also evaluated for differences with those of the general population of Germany; whether a correlation between SOC and subjective health existed was additionally examined.

**Method:**

In total, 698 male and female elite athletes, drawn from Germany's highest-level national track and field squads, and first and second division handball teams, completed a survey that included the SOC-L9 Scale and measures of subjective health, sociodemographic information, and the number of injury lay-offs experienced during the athletes' careers to date.

**Results:**

Classification tree analysis reveals six contrast groups with varying SOC scores. Several interacting factors determine the group to which an athlete belongs. Together with overuse injuries, additional factors are age, gender, and completed/not completed apprenticeship/degree. Female athletes aged between 19 and 25, who had already been subject to lay-offs due to overuse injuries, comprise the group with the lowest SOC scores. Overall, the SOC of elite athletes is slightly lower than in the general population. In accordance with other studies, a stronger SOC is also correlated significantly with better global subjective health.

**Conclusion:**

The identification of contrast groups with varying SOC scores contributes to the development of more targeted salutogenetic health promotion programs. Such programs would ideally include learning modules pertaining to coping with overuse injuries, as well as social support systems aiming to effectively combine education and elite sport.

## Introduction

Excellent health is essential for long-term, high performance levels in sports. At the same time, an athlete's health is always at risk. In addition to the common stressors of life, elite athletes are confronted with numerous sports-specific stressors. For example, they have to deal with high training loads and the physical demands of elite-level competition; management of injuries or illnesses; and coping with role conflicts generated by the differing expectations of sports organizations, families, and educational establishments. Therefore, staying healthy represents a huge challenge. From a salutogenetic perspective, elite athletes have to successfully cope with the state of tension created by these stressors in order to generate health. According to the medical sociologist Aaron Antonovsky, health is considered as a position on a health ease/disease continuum and a movement in the direction towards health [1]. In this regard, an important factor in successfully responding to stressors and staying healthy is one's sense of coherence (SOC).

SOC reflects a person's view of life and enables people to consider their external and internal resources, to identify and mobilize them, to promote effective coping by finding solutions, and to resolve tension in a health-promoting manner [2]. SOC is defined as “a global orientation that expresses the extent to which one has a pervasive, enduring though dynamic feeling of confidence that (1) the stimuli deriving from one's internal and external environments in the course of living are structured, predictable, and explicable; (2) the resources are available to one to meet the demands posed by these stimuli; and (3) these demands are challenges worthy of investment and engagement” [1].

When confronted with a stressor, a person with a strong SOC will be motivated to cope (meaningfulness) and believe that the situation may be understood (comprehensibility) and that resources to cope are available (manageability) [3]. To date, research on health in elite sport has mainly focused on biomedical perspectives, although many recent studies with non-athlete populations have demonstrated that a strong SOC promotes resilience and the development of a positive subjective state of health, especially mental health [2]. The relationship between SOC and perceived health is manifested in study populations regardless of age, gender, ethnicity, nationality, or study design. There are also strong positive correlations between SOC and different measures of well-being, as well as negative correlations with distress, maladaptation, depression, and subjective complaints [2]. Longitudinal studies employing the SOC scale reveal a relatively high degree of predictability of future health in both the short and long term. For example, a strong SOC was the most important predictor of differences between employees with serious “burnout,” and those without burnout, in a 10-year follow-up study [4]. A strong SOC was also associated with reduced risk of psychiatric disorders in a 19-year follow-up study [5].

For elite athletes in particular, who we define as athletes competing at least at the top level nationally, a high SOC should be considered a central factor for maintaining both health and performance levels in sports. Considering both the role of the SOC as a key health resource, and the extreme strain engendered by intense exercise and competition, it appears intuitive to assume that athletes with a low SOC will have serious difficulties staying healthy and being able to perform. Although there exist many studies pertaining to several aspects of SOC in different populations (and in different countries), there has been, to our knowledge, no research on elite athletes' health in this field. The only study conducted with an elite athlete population focused on the relationship between mental skills in Swedish elite athletes and the concepts of locus of control (LOC) and SOC, but not on aspects of health [6]. In light of the existing deficit in research, several theoretical considerations will first be made regarding the possible characteristics and development of SOC in elite athletes.

According to Antonovsky's theoretical assumptions, SOC develops during childhood and adolescence and stabilizes around the age of 30 [1]. In order for a strong SOC to develop, people must continually have coherent life experiences. The strength of a person's SOC, according to Antonovsky's theory [3], is shaped by different kinds of life experiences characterized by consistency, underload-overload balance, and participation in socially valued decision-making. The extent of such experiences is underpinned by many factors, ranging from one's position in society to one's culture, vocation, and family structure. Additional factors can also play a major role in a person's life experience, such as gender, ethnicity, chance occurrences, and even genetics.

It has thus far been assumed that the strength of an individual's SOC is related to their age and gender. A recent study, with 43,598 respondents, underlines the fact that males have a stronger SOC than do females. In terms of age, a stronger SOC is also observable in older age groups [7]. Both gender and age are therefore taken into consideration when calculating standard values for certain population groups [8]. Considering the dynamic nature of the development of SOC, it should be stated that SOC is not as stable as Antonovsky assumed. Evidence shows that SOC tends to increase with age and that the level of SOC achieved in adulthood is most relevant in terms of its stability. Particularly among individuals with an initially high SOC, SOC is more stable than it is in individuals with a lower initial SOC [9].

### Theoretical Considerations on the Development of Elite Athletes' SOC

Considering that SOC “is partly determined by an individual's position in the social structure and partly by current work conditions, social network, and support, rather than predominantly by experiences made in his early life” [10], [11], it may be expected that the social context of elite sport influences the strength of SOC. In the course of socialization occurring via elite sports participation, athletes are increasingly bound to the sport system. The life of an elite athlete is highly structured, through training and competition commitments, and adherence to the formal structures of elite sport. Sport networks comprised of coaches, physicians, therapists, and managers teach athletes appropriate health management in addition to the basic moral and normative concepts of elite sport [12]. Furthermore, athletes are exposed to specific health risks, principally various kinds of sports injuries.

It appears that an initially high SOC is required in order to manage the demands of elite sports and that repeated, successful management of health problems contributes to a stabilization of, or even an increase in, SOC. This is true particularly in terms of subsequent athletic success in competitions and the social recognition that stems from such efforts. This argument is supported by studies of athletes in college student populations. Athletes in a large Japanese college sample possessed a higher SOC score than did non-athletes. Furthermore, the results suggest that engaging in sporting activities for several successive years enhances SOC [13]. Another study, based on a small, non-elite sample of US college varsity athletes and college non-athletes, also reveals higher SOC scores in the athlete subpopulation [14].

Despite the above discussion, it should also be noted that SOC can be weakened. Traumatic life events as well as the appearance of severe health problems [15] can decrease SOC in both genders [16]. In the context of elite athletes, injuries have to be categorized as critical life events that place high demand on psychological ability to cope [17], [18]. In a study on the quality of life of US elite collegiate athletes, for example, injuries which required a hiatus from athletics had a negative effect on all indices of quality of life [19]. Elite athletes do not experience injury lay-offs only owing to traumatic injuries; as a result of discrepancies between stress and individual resilience, over-training syndrome and overuse injuries can also occur, sometimes leading to chronic symptoms and enforced training and competition absences. Accordingly, it may be assumed that a strong SOC can be weakened when athletes repeatedly experience instability in their athletic career and a lack of control over their health due to risk of injury. One can therefore also assume that there are certainly elite athletes with a low SOC, who do not possess the optimal conditions for coping with further elite athletic demands.

With respect to the foregoing discussion, we hypothesize that elite athletes will have a higher mean SOC than age-matched controls, but that there will still exist groups of athletes with particularly low SOC scores. In addition, we expect a strong correlation between SOC and subjective health in athlete populations.

### Objective

In summary, the primary objective of the present study is to investigate SOC in a sample of elite athletes focusing on three aspects: determination of SOC in a sample of German handball players and track and field athletes versus the general population; assessing the relationship between SOC and subjective health; and identifying subsets of athletes with either particularly high or low SOC via the method of classification tree analyses (main study objective). Therefore, the question is raised as to whether there exist subsets of elite athletes with notably different SOC values and, if so, which variables predict such differences. In addition to the influence of general sociodemographic and sport-specific characteristics, the role of injuries as a specific type of critical life event in an athlete's career progression is of major interest.

## Materials and Methods

### Study Design

Analyses are based upon data drawn from a larger multi methodical study pertaining to health management in Germany's elite handball and track and field teams. In the quantitative section of the study, a cross-sectional survey was conducted with a questionnaire covering health-related topics including SOC, subjective health, sports injuries, attitudes towards health, risk and pain, and sociodemographic and sports-specific characteristics.

### Ethics Statement

Our research was approved by the Institutional Review Board of the Faculty for Economics and Social Sciences. We adhered to the principles expressed in the Declaration of Helsinki. All participants were informed in writing about the study's aims and procedures, the confidential treatment of the data, voluntary nature of participation, and use of anonymous data in analyses. Informed consent was implied through the act of completing and returning the questionnaire. All data obtained was anonymized before it was received and therefore not connected to the athletes' personal information when entered in SPSS.

### Participants and Procedure

German male and female elite athletes meeting the following criteria were included in the study: (a) engaged in handball or track and field, (b) member of one of the highest-level national squads (i.e., A, B, C to D/C squad) and/or (c) competing in the first or second division of Germany's professional handball league. The study was supported by the National Handball Federation, the National Handball League and the National Track and Field Federation. The survey was administered in 2006. Due to sports-specific organizational differences, it was necessary to distribute the questionnaire in the following manner. (1) Track and field athletes were contacted by mail via an existing mailing list comprising all of the squad members. In total, 328 of 559 athletes completed and returned the questionnaire (58.50%). (2) For the handball players, a complete mailing list did not exist; thus, contact was made via their home teams: packages containing sealable questionnaires were sent to all 30 premier league teams and to the best 26 teams in the second division (either to the headquarters of the home teams or directly to team physicians). In total, 395 players, from 43 different teams, responded. We approximated a response rate of 50.38% based on an estimated 14 German players within each of the 56 contacted teams. 25 of the total of 723 male and female athletes who responded did not complete the SOC section and were therefore excluded from the analysis. The final study population consisted of 698 elite athletes (380 handball players and 318 track-and field athletes) with an average age of 22.85 years (see Table 1). There are slightly more male athletes (58.8%) in our sample. 36.7% of respondents consider themselves professional athletes, who make their living from their sport. 55.3% of respondents attained the highest level of school education available in Germany'; 19.8% still attend school. 34.4% have completed vocational training or a tertiary degree, and 18% currently attend university. The majority of respondents are single (85.8%). Athletes' average weekly training load amounts to 13.95 hours (season and off-season) with an average of 28.2 competitions entered per season. Athletes had experienced an average of 4.81 injury lay-offs due to traumatic injuries and 3.17 injury lay-offs due to overuse injuries, during their entire career.

**Table 1:** Sociodemographic Data and Descriptive Statistics for Health Related Variables (N = 698).

Category	Subcategory	Absolute (N)	%	M (SD), [Range]
Sex	Female	283	40.5	-
	Male	410	58.8	-
	Missing	5	0.7	
Age	Valid	674	-	22.85 (5.41), [14]–[41]
	Missing	24	-	
Education	Student (No Degree yet)	138	19.8	-
	University Entrance Degree (*Abitur*)	386	55.3	-
	Secondary School Certificate (*Mittlere Reife*)	126	18.1	-
	Secondary Modern School (*Hauptschule*)	21	3.0	-
	No degree	5	0.7	-
	Missing	22	3.2	-
Family status	Single	599	85.8	-
	Married	80	11.5	-
	Missing	19	2.7	-
Vocational Status	School Student	140	20.1	-
	University Student	131	18.8	-
	Apprenticeship	13	1.9	-
	Completed Apprenticeship/University Degree	242	34.4	-
	No Apprenticeship/University Degree	12	1.7	-
	Missing	160	22.9	-
Type of Sport	Handball	380	54.4	
	Track and Field	318	45.6	
Professional Athlete	Yes	256	36.7	-
	No	421	60.3	-
	Missing	21	3.0	-
Performance Level	A-, B-Squad/Premier league/National team (Level 1)	367	52.6	-
	C-, D/C-Squad/Second division league (Level 2)	321	46.0	-
	Missing	10	1.4	-
Career Length in Elite Sports (Years)	Valid	676	-	6.94 (4.66), [1]–[26]
	Missing	22	-	
Training in Hours/Week in Past Season	Valid	684	-	13.95 (4.86), [2]–[36]
	Missing	14	-	
Competitions in Past Season	Valid	664	-	28.2 (16.67), [1–90]
	Missing	34	-	
Traumatic Injuries: Lay-offs >1 Week	Valid	667	-	4.81 (5.09), [0–37]
	Missing	31	-	
Overuse Injuries: Lay-offs >1 Week	Valid	668	-	3.17 (5.26), [0–46]
	Missing	30	-	
Global Subjective Health	Valid	692	-	3.26 (0.73), [1]–[4]
	Missing	6	-	
Subjective Complaints (Score from 0 to 32)	Valid	680	-	4.00 (4.05), [0–21]
	Missing	18	-	
Current Competition Lay-off due to Injury or Illness	Yes	102	14.6%	-
	No	593	85.0%	-
	Missing	3	0.4%	-
Current Limitation on	Yes	358	44.1%	-
Athletic Performance	No	308	51.3%	-
	Missing	32	4.6%	-

### Measures

#### SOC

The SOC scale is considered a reliable, valid, cross-culturally applicable instrument measuring how people manage stress and remain healthy [20]. It is also considered a useful screening tool to identify individuals with inadequate coping capabilities who are particularly vulnerable to distress [21].

Alongside the SOC-29 and the SOC-13, the shorter SOC-L9 is increasingly being used [22]. An additional factor in favor of these various scales is the fact that values for population-representative random samples exist for comparison. To measure SOC, the Leipzig Short Scale SOC-L9 was utilized presently [23]. Here, SOC is measured one-dimensionally, and with a comparable rating, as per the 29-item scale, but is determined with less diagnostic effort. The Leipzig Short Scale contains the 9 items of the 29-item scale that correlate most closely with the original total scale. The one-dimensionality of the scale has been borne out via factor-analysis. The postulated subcomponents of SOC (comprehensibility, manageability and meaningfulness) are not therefore separately assessed, owing to their being unsatisfactorily replicated in many empirical studies [23]. This scale also exhibits good reliability in our elite athlete population (Cronbach's alpha  = 0.82).

#### Subjective health

As variables of subjective health, athletes' general perception of their health, and subjective health complaints, were also included. Global self-reported health was measured according to the HBSC studies [24]. Athletes were required to respond to the question “Would you say your health is…?” on a 4-point scale, as to whether their subjective health was poor, fair, good, or excellent. Global self-reported health is regarded as a valid predictor of mortality and future morbidity [25].

Subjective health complaints were measured by the HBSC Symptom Check List (HBSC-SCL) [26]. This includes the following 8 items: headache, abdominal pain, backache, feeling low, irritability or bad mood, feeling nervous, sleeping difficulties, and dizziness. Athletes reported, via a 5-point scale, if each symptom was experienced most days, more than once a week, about once every month, seldom, or never. Non-occurrence of a symptom corresponded to 0; occurrence most days corresponded to 4. Total scores were calculated for each athlete between a minimum of 0 and a maximum of 32 [27]. The reliability and validity of the instrument has been confirmed, and the overall scale is considered to measure one-dimensional, latent psychosomatic complaints [26]. Within our elite athlete population this scale also has acceptable reliability (Cronbach's alpha  = 0.74).

#### Variables used in the classification tree analysis

Eleven variables from three categories were incorporated into the classification tree analysis. This method will be described in more detail in the data analysis section. Table 2 displays the characteristics of the dependent and independent variables. In addition to common sociodemographic statistics such as age, gender and marital status, and educational and vocational status, sports-specific sociodemographic variables, such as type of sport engaged in, squad level, professional status, career duration, training load, and number of competitions entered were incorporated. To identify injuries as a specific type of critical life event in an athlete's career, the number of severe injury lay-offs due to overuse and traumatic injuries was assessed. According to Bird, Black, and Newton [28], traumatic injuries are those which occur due to an accident with or without an external influence such as tears (torn ligaments, ruptured muscle fiber, etc.), breaks, strains, bruises, or open wounds. Overuse injuries were categorized as physical symptoms caused by improper or excessive biomechanical stress; for example joint pain owing to enthesitis, tendinitis, or bursitis. To calculate the overall prevalence of each type of injury, an extensive list of self-developed filter questions pertaining to previous career injuries (differentiated according to location) was employed. If a filter question was answered with a “yes,” athletes were then asked how often they had been “laid off” for at least 1 week due to the injury. Athletes' answers were separated according to type of injury (traumatic/overuse): results were summed to obtain a total score.

**Table 2:** Classification Tree Analysis: Description of Dependent and Independent Variables.

**Target Variable**	**Type**	**Description**
Sense of Coherence	Interval	SOC-L9; Scale from 9 to 63. Higher values represent a stronger sense of coherence.
**Independent Variables**	**Type**	**Description**
*Sociodemographic Characteristics*		
Sex	Nominal	Male, Female
Age	Ratio	Age at time of the survey
Education	Nominal	University Entrance Degree (*Abitur*), Secondary School Degree (*Mittlere Reife*), Secondary Modern School Degree (*Hauptschulabschluss*), No School Degree (Student), Other School Degree
Family Status	Nominal	Single, Married
Vocational Training	Nominal	School Student, University Student, Apprenticeship, Completed Apprenticeship/University Degree, No Completed Apprenticeship/University Degree
*Elite Sport-Specific Sociodemographic Characteristics*		
Type of Sport	Nominal	Handball (Team Sport), Track and Field (Individual Sport)
Professional Athlete	Nominal	Full-time employment in elite sport (yes/no)
Performance Level	Ordinal	Level 1: A-/B-Squad (T&F) and National/Junior National/Premier League Team (HB), Level 2: C- & D/C-Squad (T&F) & Second Division League (HB)
Career Length in Elite Sports	Ratio	Performance-oriented participation in sport in years
Training Length	Ratio	Average length of training per week in hours
Competition Frequency	Ratio	Total number of completed competitions in the previous season
*Traumatic & Overuse Injuries in the Athletes*' *Career as Sports-Specific Critical Life Events*		
Training and Competition Lay-offs >1 Week due to traumatic injuries	Ratio	Total number of necessary training and competition lay-offs lasting longer than 1 week in the athletes' career to date due to traumatic injuries (e.g. tears, breaks, sprains, open wounds).
Training and Competition Lay-offs >1 Week due to overuse injuries	Ratio	Total number of necessary training and competition lay-offs lasting longer than 1 week due to overuse injuries (physical complaints because of too much or improper biomechanical stress, e.g. enthesitis, tendonitis, or bursitis).

### Analysis

#### Descriptive statistics and group differences

Descriptive statistics are herein based on mean, standard deviation, and range values. To determine group differences in SOC, between the elite athlete population and an age-appropriate, representative comparison group, drawn from German-speaking countries [22], one-sample t-tests were calculated for both males and females. The confidence interval was set at 95%, and alpha at 0.05. Moreover, effect sizes were calculated using Cohen's *d*.

#### Correlation calculations

Correlations between SOC, subjective health, incidence of subjective symptoms, and the necessity of an injury-induced lay-off from sport were assessed, where only correlation coefficients with a significance of *p*<.001 were considered relevant, in light of the large sample.

#### Classification tree analysis

Classification tree analysis is considered a promising research tool for identification of at-risk populations in public health research and outreach [29]. However, this method is not yet commonly used in public health or sports science. Classification tree analysis allows for exploratory identification of contrast groups, based on potential factors influencing the development of SOC. Compared to other multivariate methods, classification tree analyses have the following three main advantages [30]–[32]: the ability to separate a population into subgroups whose members share common characteristics; simultaneous treatment of interactions among independent variables; and high flexibility, conferred by handling a variety of variable types simultaneously (continuous, ordinal, or nominal) and not requiring the stringent theoretical and distributional assumptions of more traditional methods (such as cluster analysis, discriminant analysis, and regression models).

One commonly used classification tree technique is the so-called Chi-Squared Automatic Interaction Detector (CHAID), which was originally utilized as a data mining technique in the field of marketing, in order to segment large samples into homogenous subgroups. Owing to the successful implementation of data mining techniques in business-related contexts, these techniques have increasingly been used to analyze patterns in health care and rehabilitation contexts [32], clinical and surgical settings [33], and epidemiology [30]. Given the complex nature of the development of SOC, with potential interactions between several different independent variables, classification tree analysis employing the exhaustive CHAID algorithm is an appropriate way to identify and characterize contrast groups in larger samples. It is important to note that, in addition to exhaustive CHAID, there are several varieties of decision tree method. These methods, including CHAID, Quick, Unbiased, Efficient Statistical Trees (QUEST) and Classification and Regression Trees (C&RT), refer to various algorithms which differentially affect the way in which the tree is “grown” [29]. A recent study modeling landslide hazard via decision trees demonstrated that the exhaustive CHAID algorithm had the highest degree of classification accuracy [34].

The present classification tree analysis was performed via SPSS for Windows (SPSS, Ver. 19.0, Chicago, IL., USA) using the method of exhaustive CHAID. Exhaustive CHAID uses a systematic algorithm to detect the strongest association between the target variable and independent variables. At each step, the independent variable with the strongest interaction with the dependent variable is chosen. The categories of each independent variable are merged if they are not significantly different with respect to the dependent variable. Exhaustive CHAID is a modification of the CHAID algorithm that examines all possible splits for each dependent variable, thereby effecting greater precision [35], [36]. The starting point of the analysis is the so-called “root node” at the top of a tree model, which contains the whole data set of the target variable. If a node splits to form part of a new level, it is referred to as a “parent node.” Nodes splitting even further from the parent node are termed “child nodes.” Each node at the end of a branch is labeled a “terminal node.” Terminal nodes represent the identified contrast groups. Nodes are represented by boxes including the mean value, standard deviation, absolute numbers, and percentage of the sample contained therein. Above each node, the name of the most significant predictor variable, the *p*-value, chi-squared value, and degrees of freedom are illustrated.

The SOC-L9 score was employed as the target variable, with possible outcomes ranging between 9 and 63. The 11 predictor variables included in the analyses are displayed in Table 2. For continuous dependent variables, data are automatically divided into approximate deciles and used as ordinal variables in the analysis [36]. The following parameters were chosen in order to “grow” the tree: a maximum tree depth of 3, a minimum parent node size of 100, a minimum child node size of 40, and a significance threshold of 0.05 for splitting. Because the dependent variable (SOC) is neither nominal nor ordinal, the overall misclassification risk in the tree cannot be calculated using the correctly classified individuals. The misclassification risk was therefore given by calculating the proportion of variance explained through the model using the risk estimators and the total variance of the root node [36].

## Results

### SOC of Elite Athletes in Relation to the General Population and Subjective Health

As can be seen in Table 3, elite athletes' mean SOC scores are slightly lower than are those of the general population group (aged 17 to 40). With alpha set at 0.05, the one-sample t-test reveals that mean differences (female: 1.86; male: 0.97) are statistically significant (female: *t* = −3.95 (282), *p*<0.001 and male: *t* = −2.83 (409), *p* = 0.005). The 95% confidence interval indicated that the populations' mean difference is likely to fall within −2.79 and −0.93 for females and −1.65 and −0.3 for males. However, the differences have little practical relevance considering the effect sizes (female: Cohen's *d* = 0.24 and male: Cohen's *d* = 0.13).

**Table 3:** Sense of Coherence in Comparison to Population Representative Norm Values.

**Athletes SOC-L9**	**N**	**M**	**SD**		**Median**
Total Sample^1^	698	48.85	7.41		50
Female	283	47.82	7.93		49
Male	410	49.52	6.96		51
**German Norm Sample SOC-L9 (Hannöver et al., 2004)**	**N**	**M**	**SD**		**Median**
Age Group 17**–**40: Female	1022	49.68	7.88	(p = 0.000)*	51
Age Group 17**–**40: Male	1012	50.49	7.77	(p = 0.005)*	51

1 This study's sample of athletes has an average age of 22.85. 88.1% of the athletes surveyed are aged between 17 and 40. In the German general population sample SOC-L9 (cf. Hannöver et al. 2004), only gender-specific values exist for the age group 17–40.

*Significant at *p*<0.05. One-sample t-test calculated for both genders and for both general population samples. Confidence interval 95%.

To test the correlation between SOC and subjective health, which has been previously documented in many populations, correlations between SOC scores and global assessment of current health, incidence of subjective complaints, and necessity of an acute injury lay-off were calculated. The correlation coefficients displayed in Table 4, for the elite athlete population, confirm the expected correlations between SOC and general health perception, as well as subjective health complaints. However, the correlation coefficients have low values.

**Table 4:** Correlation Between Sense of Coherence and Indicators of Subjective Health.

Scale	Sense of Coherence SOC-L9
Global subjective health	0.281*
Subjective complaints (Score)	−0.384*
Current competition lay-off due to injury or illness	−0.072
Current limitation to athletic performance ability due to injury/illness	−0.135*

*The correlation is significant at the level of 0.01 (2-sided).

### Contrast Groups with Varying SOC-Scores: Predictors Using Classification Tree Analysis

**Figure 1:**
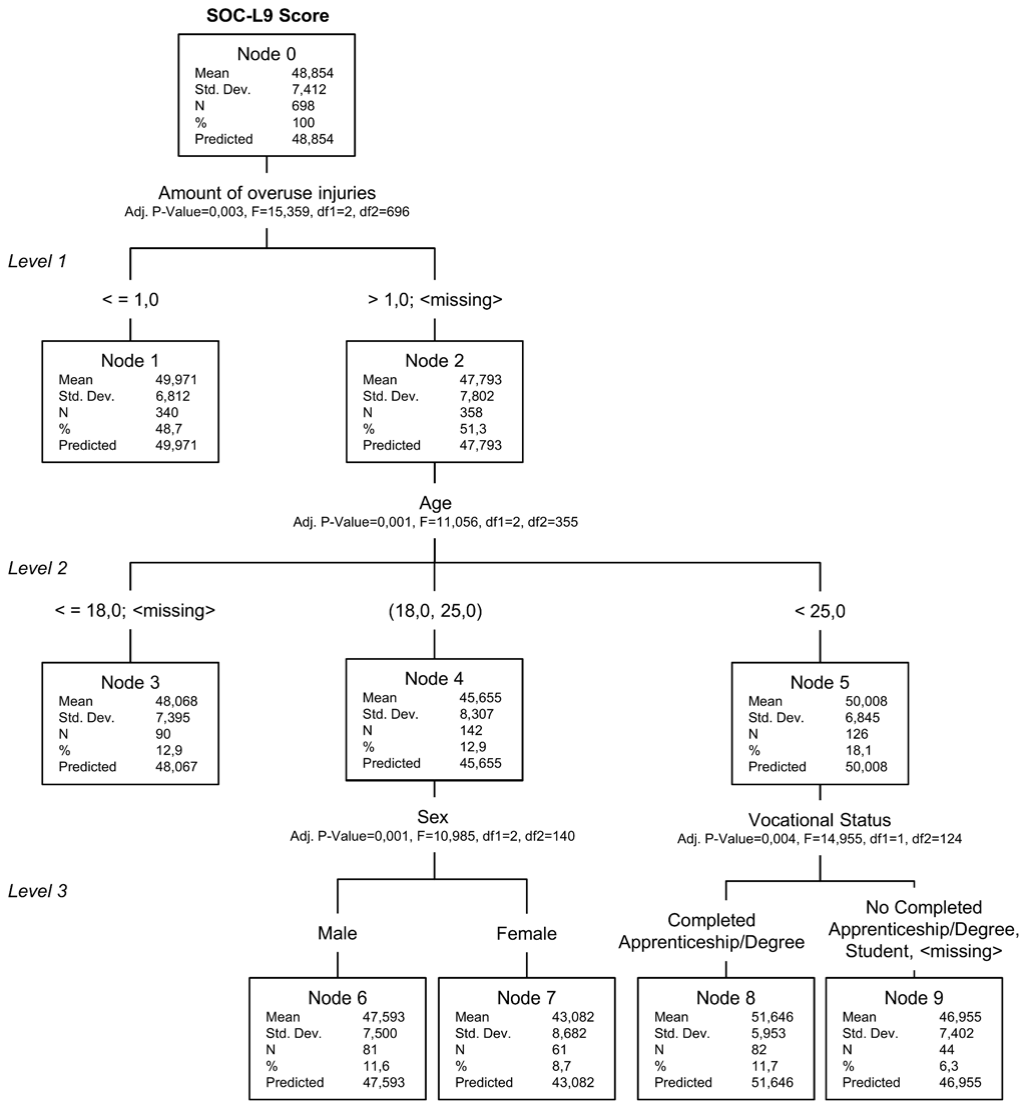
Classification Tree of Predictors Associated with SOC.

On the basis of the most significant factors, which split the data set, the tree model depicted in Figure 1 was constructed. The tree model demonstrates that the SOC value is predicted by four interacting factors from three different levels. The variable with the strongest influence on SOC is the number of lay-offs due to a severe overuse injury (level 1). Age has the second strongest influence (level 2). In the 19 to 25 years age group, gender is the next best predictor (level 3), and in the age group of athletes older than 25, SOC score is significantly influenced by vocational status (level 3). All the other variables, for example the type of sport participated in or level of school education, have no significant influence on SOC. The proportion of variance explained by the classification tree model is 9.12%, which dictates that there are additional influencing variables that were not included in our study.

The tree model indicates that the age of athletes is only a significant factor when athletes have experienced two or more overuse injury lay-offs. Gender and vocational status are significant influencing factors on SOC only in specific age groups. For example, a gender difference in the 19 to 25 year-olds was only observable among those athletes who had already experienced multiple injury hiatuses owing to overuse injuries. Regarding the terminal nodes, six different contrast groups with specific SOC scores and characteristics were identified (Table 5).

**Table 5:** Classification Tree Analyses: Gains Chart.

Knot	Description	N	Size	SOC-L9 Mean (SD)
8	Two or more severe overuse injury lay-offs, 26 years or above, completed apprenticeship/university degree	82	11.7%	51.65 (5.95)
1	Less than one severe overuse injury lay-off	340	48.7%	49.97 (6.81)
3	Two or more severe overuse injury lay-offs, 18 years or below	90	12.9%	48.07 (7.39)
6	Two or more severe overuse injury lay-offs, 19–25 years, male	81	11.6%	47.59 (7.50)
9	Two or more severe overuse injury lay-offs, 26 years or above, no completed apprenticeship/university degree	44	6.3%	46.96 (7.40)
7	Two or more severe overuse injury lay-offs, 19–25 years, female	61	8.7%	43.08 (8.68)

1. The group with the highest SOC score, of 51.65, consists of athletes who experienced at least two severe overuse injury lay-offs during their career, who are older than 25, and who have already completed their apprenticeship/degree. In total, 11.7% of the study subjects belong to this group (node 8).
2. The largest identified group has the second highest mean SOC score, of 49.97. This group is characterized by athletes who have never, or only on one occasion, experienced a severe overuse injury lay-off. 48.7% of the study subjects belong to this group (node 1).
3. The third-highest SOC score (48.07) occurred for those athletes who have experienced at least two 1-week lay-offs due to overuse injuries, and who are under 19 years of age. 12.9% of the study subjects belong to this group (node 3).
4. Another group consisted of male athletes aged between 19 and 25 years who had an overuse injury-related lay-off at least twice. The mean SOC for this group was the third lowest at 47.59, and where this group size comprised 11.60% of the total respondents (node 6).
5. The group with the second-lowest SOC scores comprised athletes who have had at least two overuse injury lay-offs during their career, are older than 26 years, and have not completed an apprenticeship or tertiary degree. 6.3% of the respondents belong to this group, with a mean SOC score of 46.96 (node 9).
6. The lowest mean SOC score, by some distance (of 43.08), was seen in the group characterized by female athletes aged between 19 and 25 years, who have experienced two or more severe overuse injury hiatuses during their career. 8.7% of the total respondents belong to this group (node 7).

Comparison of the contrast group mean SOC scores with the total mean SOC scores for the athlete population (cf. Table 3) indicates that only nodes 8 and 1 have above-average SOC scores. The mean for nodes 6, 9, and 7 are all less than the total sample mean, and are less than the mean for the female athletes alone.

## Discussion

From a salutogenetic perspective, successfully coping with stressors is highly relevant for both elite athletes' health and their performance in sports. It is evident that SOC plays a fundamental role in this regard. The main purpose of the study was identification of contrast groups with high and low SOC scores, using the promising method of classification tree analysis, based on a large population of Germany's elite handball and track and field athletes. Analyzing elite athletes contributes to our understanding of how extreme physical and psychological demands in a highly competitive social context affect an individual's SOC. Accordingly, athletes' SOC scores were additionally compared with SOC scores in the general population. In order to examine the health-related relevance of an individual's SOC to coping with physical and psychological stressors in elite sports, the correlation between SOC and subjective health was also analyzed.

### SOC of Elite Athletes in Relation to the General Population and Subjective Health

Our analysis demonstrates that the SOC of elite athletes is slightly lower than that of the general population. Although these differences are significant, they should not be over-interpreted due to the small effect sizes and large study population. Nevertheless, this finding is surprising considering that good physical and mental health is a key factor for achieving high performance levels in sports, and also that a high SOC is normally associated with above-average health. It can therefore be argued that elite athletes do not have better preconditions for good health than do the general population. Furthermore, the athlete population exhibits a similar SOC to clinical populations, in which patients with psychological challenges score lower than do random samples [2]. The generally lower SOC scores observed in elite athletes may be explained by the instability of their athletic careers, engendered by constant pressure to perform in combination with a permanent risk of injury. Sports injuries, which are accompanied by a decrease in athletic performance, can lead to a perceived lack of control over life and challenge athletes' overall identity. Additionally, the need to permanently exist on the boundary between overload and optimal performance can lead to a discrepancy between stress and individual resilience.

Our findings differ from those of other studies of college athletes, who had higher SOC values than did college non-athletes [13], [14]. This could be explained by the greater career length and number of stressors that manifest at an elite level, compared to average college athletes. Health problems, particularly due to overuse, often pose huge career risks to the future career development of elite-level athletes. Furthermore, athletes have to cope with demanding environments (e.g. sponsors, journalists, and officials) and many different stressors (e.g. competing for positions in national teams, contract negotiations, and attaining a professional education for life after their sporting career finishes).

Our findings confirm that SOC and subjective health also correlate in elite athletes. As in other cross sectional studies with different populations across several countries, a strong SOC positively correlates with better global subjective health and negatively correlates with subjective health complaints [2], [7], [37]. A stronger SOC also negatively correlates with subjective performance limitations due to injury and illness. This could be interpreted as further evidence that SOC is a general health-related source of resistance [2].

### Contrast Groups with Varying SOC Scores

To our knowledge, classification tree analysis has not been applied to SOC research thus far. As our results show, this method is useful for identifying subpopulations at risk for developing future biopsychosocial health problems. Although our study population should to be regarded as very specialized, we identified contrast groups with higher and lower than average SOC scores. According to our results, classification tree analysis can help us better understand the simultaneous interaction of different factors which influence SOC. Several interacting factors determined the group to which an athlete belonged; in addition to overuse injuries, age, gender, and whether athletes had completed their vocational training or educational degree were also factors. This is in line with previous research demonstrating that SOC relates to age and gender [7], the appearance of severe health problems [15], [16] and professional career events [11]. Nevertheless, as the identification of contrast groups demonstrates, these factors only play a role in certain instances. When compared to previous studies, this provides new insight into the interplay which exists between several factors when predicting SOC.

A direct link exists between SOC and previous overuse injury experiences in the group with the second highest overall score. This could be because these athletes have had very few overuse injuries. Here, we see neither large deviations from the German general population, for men or women, nor differences in the influence of age, gender, or apprenticeship status. That overuse injury lay-off experiences have an influence on SOC is consistent with salutogenetic theory: an initial, already well-established SOC is further stabilized if no incidents occur that endanger the functionality of general resistance resources [3]. The successful management of tension engendered by the demands of the elite sports system, without experiencing stress and developing overuse injuries, indirectly stabilizes SOC through the consolidation of generalized resistance resources. In this regard, the social support system plays an important role. Both training control systems designed to minimize overloads, and experienced team doctors who detect and successfully treat early symptoms of overuse, can contribute to the development and maintenance of a strong SOC.

Our results further demonstrate that experiencing multiple overuse injury lay-offs is associated with a decrease in SOC. However, this only pertains to certain constellations of age, gender, and educational degree status. Among athletes who have experienced multiple overuse injury lay-offs during their career, those aged 18 and below had a greater SOC than 19–25-year-old athletes. This may be owing to the cumulative impact of overuse injuries over the course of an athlete's entire career: if an injury occurs during an elite athlete's prime (typically their mid-20s), the impact is more significant than if it occurs during the earlier stages of their career. Moreover, adolescents are more focused on the “here and now,” which dictates that the possible consequences of overuse injuries have probably not yet been fully realized. Furthermore, given the fact that many athletes under 18 still attend school, the biographical fixation on elite sports is probably not yet as strong as it is for adult athletes.

Female athletes aged between 19 and 25 who have experienced overuse injuries are a high-risk group for low SOC scores and indeed had the lowest SOC scores in this study. In this age group, crises and career insecurities, along with overuse injuries, are felt most acutely by female athletes. In contrast to the large study of Nilsson et al. [7] who employed more common statistical methods, and where males had a stronger SOC than females, we identified gender differences only in one age group. The significantly lower SOC in females, in contrast to men in the same age group, may be because, in this age group, women's SOC scores are particularly vulnerable to overuse injuries. This corresponds with the fact that a downturn in the health of women has a stronger effect on SOC than it does for men [7]. Moreover, and in relation to the influence of life events, gender-specific differences are reported, which vary according to the type of life event [16].

Our findings also support the theoretical assumption that successfully coping with stressors leads to an increase in SOC under certain conditions. In our study, athletes aged 26 years and above, and who have completed an apprenticeship, exhibit the highest SOC scores, despite having experienced two or more overuse injuries. These athletes were obviously able to both successfully cope with overuse injuries and to successfully manage non-athletic career challenges.

In the case of professional insecurity owing to an incomplete education, older athletes who have already experienced overuse injuries have distinctly lower SOC scores, even compared to the general population. For older athletes who have not completed their education, life appears less meaningful, less comprehensible, and less manageable. Accordingly, this group is expected to have more difficulties in maintaining health than athletes with a completed education.

Antonovsky's assumption that SOC automatically increases with age does not hold true for our elite athlete population [1]. Nevertheless, the findings confirm Antonovsky's assumption that SOC develops until approximately the age of 30 and further that it can be influenced by critical life events. However, against the background of our findings it has to be assumed that in elite sports age only influences SOC development indirectly. This is in line with research demonstrating that a high SOC, rather than greater age, seems to effect stable development of the SOC [9]. Given the important influence of overuse injuries, and the role of vocational status, it can also be stated that SOC is less stable than was assumed by Antonovsky. Our data concords with a longitudinal study that reports a general lack of stability in SOC [38].

Of particular interest are the factors in the classification tree analysis that have no significant influence on SOC strength. The type of sport engaged in plays no role in our analysis, perhaps because the general principles and basic structures of the elite sports system are applicable to all athletes. For example, a set of regulations, defined competition calendar, and structured training units apply to all types of sports. In addition, career length; performance level; extent of training; frequency of competition; and professional, educational, and family status have no influence on SOC. A comparison with athletes from lower-level leagues or without squad status might lead to different results, however. Interestingly, crisis events such as frequent traumatic injuries, play no, or only a minor role, in contrast to overuse injuries. This may be due to a selection effect, however, as it can be assumed that only athletes who were able to achieve their previous level of performance after a traumatic injury participated in the study. Athletes whose traumatic injuries led to an inability to continue to engage in their sport, or resulted in a severe limitation of their ability to participate in elite sports, are unlikely to be in the sample because they ended their career prematurely. From an athletes' point of view, this could also be the result of improved knowledge and management of traumatic injuries, in terms of diagnostics, treatment strategies, and rehabilitation procedures, in comparison to overuse injuries [39]. The overall results therefore suggest that, in particular, the occurrence of overuse injuries should be considered as a critical life event for athletes because they have a profound effect on SOC strength.

### Limitations and Directions for Future Research

The study underlines the fact that identifying mutually exclusive groups, by summarizing and segmenting large datasets using exhaustive CHAID, is useful in SOC research. Nevertheless, some limitations must also be considered. As the intention of the exhaustive CHAID procedure is to identify distinct population subgroups, it does not allow for the estimation of the net effects of a single independent variable on the dependent variable, while accounting for other factors, as is the case with common regression techniques [29]. Therefore, it is not possible to predict increases or decreases in SOC, for example, in relation to the number of overuse injuries experienced. Nevertheless, and in contrast to more conventional methods of regression analysis, it is possible to model multilevel interactions and to simultaneously include more independent variables, for a more complex model that is still relatively easy to interpret [30], [32]. Due to its exploratory character, it is necessary that the present study be replicated with other samples of elite athletes in order to corroborate the predictive capacity of the model.

Another limitation of our study concerns the sample, which only consists of elite athletes drawn from two sporting disciplines. This may limit the representativeness of our results for all elite athletes. However, and at least for the sports in question, the study achieved representativeness by contacting all of the elite athletes who were active in these sports.

Regarding the interpretation of the SOC scores observed, another general problem emerged: it is not yet clear at which point SOC loses its protective effect on health and the development of severe health problems begins [2]. This complicates the interpretation of the identified groups with regard to their risk of developing future health problems. A further limitation of our study concerns its cross-sectional design, which does not allow for conclusions about causal relations to be drawn. The long-term stability of SOC in elite sports can therefore not be evaluated with the present study, nor can the question of the extent to which overuse injuries may be connected to a low initial SOC. This study also has the typical limitations characteristic of survey studies, which rely on self-report variables. Particularly in terms of assessment of the extensive medical history of injury events via questionnaire, it can be assumed that not all events will be remembered and that incomplete answers may be provided. This may be especially pertinent for questions pertaining to critical life events, where a bias can be expected leading to underestimation of the importance of these events [40].

For future research, it is recommended that longitudinal studies, examining the influence of different factors on SOC throughout an entire athletic career, be conducted. By doing so, interrelations between SOC, other general resistance resources, and health outcomes may be analyzed in detail, along with the dynamics of possible changes in athletes' SOC. Here, the impact of sports-specific critical life events, such as injuries or a career coming to an end, should be analyzed more thoroughly. In particular, time elapsed subsequent to retirement appears potentially impactful on changes in SOC. The role of SOC in successfully overcoming sports and chronic overuse injuries is also of particular interest. Overuse injuries should be measured using more recently developed and elaborate methods [41], as should the relationship between SOC, health status, and ability to perform. It is conceivable that ability to perform in training and competition is the decisive factor determining whether athletes view their lives as meaningful, comprehensible, and manageable. Follow-up studies might focus on extreme cases of highly resilient athletes, such as elite athletes with greatly above average SOC scores, who are successfully pursuing dual careers. Analysis of highly resilient athletes appears particularly promising for ascertaining what exactly confers such fortitude. Analyzing highly gifted elite athletes could therefore contribute to the general understanding of how people manage extreme stress and pressure in order to perform and remain healthy. In this context, qualitative methods employing athletes' biographies should be applied along with quantitative methods.

## Conclusion and Recommendation

Salutogenetic research on health generally adds important knowledge to the prevalent pathogenetic point of view, with its focus on the risk factors for, and causes of, specific injuries or diseases. To our knowledge, this study is the first to analyze SOC as the principal concept of salutogenetic research in an elite athlete population, and the first to use classification tree analyses to this end. Classification tree analysis should be emphasized in SOC research, because it allows for identification of sub-populations at risk for a low SOC and the attendant risk of developing future health problems. Moreover, identification of contrast groups with high and low SOC scores supports the development of health promotion programs that are more directly targeted at specific groups. Based on our study, a particular risk group for a low SOC comprises female athletes aged between 19 and 25 years who have already experienced several enforced absences due to overuse injuries. For this group specific, tailored interventions, such as general stress management courses or educational programs concerned with coping and prevention strategies are required. Furthermore, highly individualized and controlled training frameworks aimed at avoidance of systematic overload experiences should be provided. Tailored interventions are also needed for athletes older than 26 years who have had two or more overuse injury lay-offs, and who have not completed an apprenticeship or university degree. This group could be supported through individual career counseling, optional apprenticeships for post-career purposes, and more effective synchronization of educational and sportive schedules.

The present study's results have further implications for relevant stakeholders. Sports organizations, such as federations and clubs, should generally facilitate athletes to complete a dual-career, through combining education with high performance sport. They should also provide team doctors, physiotherapists and sports psychologists who have expertise in attending to overuse injuries. Injury rehabilitation processes should be highly person-centered and systematically employed to educate athletes with regard to preventive health, such that occurrence of an injury might lead to even greater resilience and SOC. Coaches could further contribute to the development of constitutional and psychosocial resistance resources by strengthening athletes' general athletic abilities and buffering the extreme social pressure situations which occur in sports. Concerning reduction of risk of chronic or recurring injuries, coaches should in particular try to temper the social expectations that lead to training and competing despite the presence of acute health problems. Albeit that initial studies demonstrate the positive effects of salutogenetic concepts on inpatient psychosomatic treatments and health outcomes [42], the effects of different salutogenetic health promotion interventions in elite sports have not yet been evaluated. It is hoped that future research will address this knowledge gap.
